# Identification of genes expressed in cultures of *E. coli *lysogens carrying the Shiga toxin-encoding prophage Φ24_B_

**DOI:** 10.1186/1471-2180-12-42

**Published:** 2012-03-22

**Authors:** Laura M Riley, Marta Veses-Garcia, Jeffrey D Hillman, Martin Handfield, Alan J McCarthy, Heather E Allison

**Affiliations:** 1 Microbiology Research Group, Institute of Integrative Biology, University of Liverpool, BioSciences Building, Crown Street, Liverpool L69 7ZB, UK; 2 Program in Molecular Structure & Function, The Hospital for Sick Children, 555 University Avenue, Toronto, ON M5G 1X8, Canada; 3 Oragenics, 13700 Progress Blvd, Alachua, FL 32615, USA

## Abstract

**Background:**

Shigatoxigenic *E. coli *are a global and emerging health concern. Shiga toxin, Stx, is encoded on the genome of temperate, lambdoid Stx phages. Genes essential for phage maintenance and replication are encoded on approximately 50% of the genome, while most of the remaining genes are of unknown function nor is it known if these annotated hypothetical genes are even expressed. It is hypothesized that many of the latter have been maintained due to positive selection pressure, and that some, expressed in the lysogen host, have a role in pathogenicity. This study used Change Mediated Antigen Technology (CMAT)™ and 2D-PAGE, in combination with RT-qPCR, to identify Stx phage genes that are expressed in *E. coli *during the lysogenic cycle.

**Results:**

Lysogen cultures propagated for 5-6 hours produced a high cell density with a low proportion of spontaneous prophage induction events. The expression of 26 phage genes was detected in these cultures by differential 2D-PAGE of expressed proteins and CMAT. Detailed analyses of 10 of these genes revealed that three were unequivocally expressed in the lysogen, two expressed from a known lysogenic cycle promoter and one uncoupled from the phage regulatory network.

**Conclusion:**

Propagation of a lysogen culture in which no cells at all are undergoing spontaneous lysis is impossible. To overcome this, RT-qPCR was used to determine gene expression profiles associated with the growth phase of lysogens. This enabled the definitive identification of three lambdoid Stx phage genes that are expressed in the lysogen and seven that are expressed during lysis. Conservation of these genes in this phage genome, and other Stx phages where they have been identified as present, indicates their importance in the phage/lysogen life cycle, with possible implications for the biology and pathogenicity of the bacterial host.

## Background

Shigatoxigenic *Escherichia coli *(STEC) cause disease in humans following colonisation of the intestinal tract [1]. These infections are often serious, presenting with severe diarrhoea accompanied by haemorrhagic colitis. Downstream sequelae such as haemolytic uraemic syndrome (HUS) and thrombotic thrombocytopenic purpura (TTP) can be fatal [2,3].

The principle defining virulence determinant of all STEC strains is the production of Shiga toxin (Stx), also known as verocytotoxin (VT) or Shiga-like toxin (SLT) (1), of which there are two distinct forms, Stx1 and Stx2 [4]. Two variants of Stx1 have been identified [5,6], whilst Stx2 is heterogeneous, with some variants more frequently associated with serious STEC outbreaks [1,7]. The *stx *genes are carried by temperate lambdoid bacteriophages, which enter either the lytic or the lysogenic pathways upon infection of a bacterial cell [8-10]. Any bacteriophage encoding Stx is termed an Stx phage, and there is much genotypic and phenotypic diversity within this loosely-defined group [11]. Integrated Stx phages may exist in the bacterial chromosome as inducible prophages, or their residence within a host cell may facilitate recombination events leading to the loss of prophage sequences, resulting in uninducible, remnant Stx prophages within the lysogen chromosome [12]. The *stx *genes are located with genes involved in the lytic cycle; hence Shiga toxin expression occurs when Stx phages are induced into this pathway [11,13].

Stx phages possess genomes that are generally ~50% larger than that of the first described lambdoid phage, λ itself, and ~74% of Stx phage genes have not been definitively assigned a function [11]. Genes that are essential for the Stx phage lifestyle are carried on approximately 30 kb of DNA [14], whilst the entire genome is ca 60 kb in size in most cases [11,15,16]. The impact of Stx prophage carriage on the pathogenicity profile or biology of the host, beyond conferring the ability to produce Shiga toxin, has remained largely unexplored and it can be suggested that the accessory genome of Stx phages is likely to encode functions for which there has been positive selection [11].

In this paper, we describe the use of proteomic-based protein profile comparisons and Change Mediated Antigen Technology™ (CMAT) (Oragenics Inc.) [17] to identify Stx phage genes that are expressed during the lysogenic pathway. An *E. coli *lysogen of Φ24_B_::Kan, in which a kanamycin-resistance cassette interrupts the *stx_2_A *gene [18] of a phage isolated from an *E. coli *O157:H7 disease outbreak strain, was subjected to both CMAT and two dimensional polyacrylamide gel electrophoresis (2D-PAGE) analyses of the expressed proteome. The Φ24_B _::Kan genome is 57.6 kb in size and is identical in all aspects to its wild-type parental phage other than the *stxA *gene interruption [14,18]. The majority of genes and coding sequences (CDS) carried by Φ24_B _are simply annotated as hypothetical [GenBank: HM_208303]. Bacteriophages tightly regulate expression of their genes involved in maintenance of lysogeny versus replication of viral progeny, and the differentiation of gene expression associated with each state needed to be carefully determined in order to definitively associate expressed proteins and their genes with either the temperate or the lytic cycle.

## Results

### The rate of spontaneous lysis in an *E. coli *MC1061(Φ24_B_) culture at different stages of growth

Spontaneous induction, defined as the induction of prophages from lysogens in the absence of an applied stimulus [19], occurs constantly in a proportion of the lysogen population in any culture, and this could seriously interfere with the differentiation of gene expression between lytic and lysogenic states. In this study, it was necessary to determine culture conditions under which the number of spontaneous induction events was low whilst the cell density was high, enabling the consistent harvesting of sufficient amounts of cell-associated protein for downstream analyses. Lysogen cultures were sampled at hourly intervals beginning two hours post inoculation, and the c.f.u. ml^-1 ^and p.f.u. ml^-1 ^determined. The lowest ratio of infective phages to cells, 1:50, occurred at both 2 h and 3 h of lysogen growth. However the c.f.u. ml^-1 ^during these times was relatively low; OD_600 _= 0.184 (± 0.003) and OD_600 _= 0.651 (± 0.008), respectively. The ratio of phage to host cells increased sharply after 4 h of growth, before dropping after 5 h to 1:33 (OD600 = 1.192 [± 0.011]). The ratio of phage to cells in the culture remained stable at 1:33 through to 6 hours of growth. Lysogen growth conditions were therefore standardised for MC1061 (Φ24_B_) at 5-6 hours when the cells were grown to an OD_600 _of 1.2-1.3.

### Phage-encoded, lysogen-culture gene expression identified by CMAT

**Figure 1:**
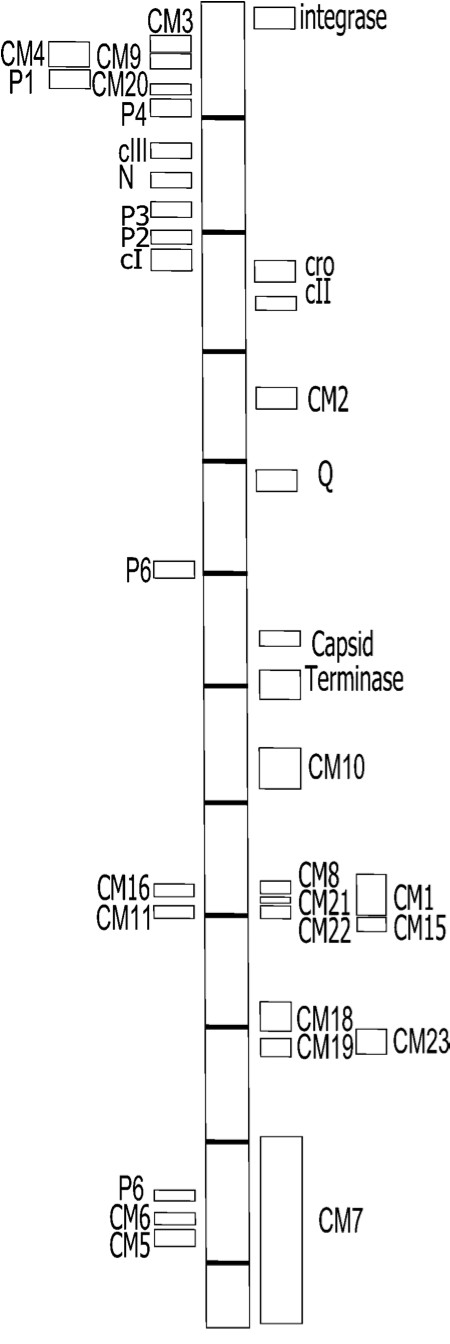
**Schematic representation of the Φ24_B _genome**. Squares symbolise the locations of the CMAT and PAGE CDS identified as well as some of the essential genes involved in the life cycle of the phage. - represents 5 kb. For further details on the gene identities see Tables 1 & 2.

A total of 13,519 clones were subjected to CMAT primary screening, and taking efficiency of the library into account, this equates to a 3.3x coverage of the phage genome. Of these, 330 were identified by the lysogen-specific antiserum and chosen for further analyses and secondary screening. After two rounds of secondary screening, 250 clones were removed from the study and PCR analysis of the remaining 80 clones demonstrated that 46 possessed vector DNA only. The remaining 34 recombinant transformants produced a peptide recognised by antibodies in the lysogen specific antiserum. The cloned inserts were sequenced, and the DNA sequences translated in all six possible reading frames. Twenty-three of the clones possessed sequences from twenty different Φ24_B _CDS (Table 1, Figure 1). The remaining eleven clones did not align with any Φ24_B _-encoded CDS, although six did possess non-coding regions of the phage genome. The other five clones contained plasmid DNA only.

**Table 1:** CDS identified by CMAT and location on the Φ24_B _genome

Clone	Alignment to Φ24_B _genome	Aligned CDS	Possible gene
CM1	39370-39772	38090-40027	*tspS*
CM2 + CM14	17489-18104	17559-18086	*dam*
CM3	2523-2185	a: 2378-2286	
		b: 2507-2379	
CM4	3025-2375	a: 2545-2375	
		b: 2812-2711	
		c: 2911-2840	
CM5	54385-53866	53693-53866	
CM6	53690-53235	53482-53297	
CM7 + CM13	55160-55667	49148-57571	
CM8	38754-39248	38460-38954	
CM9	2542-2940	2248-2646	
CM10	35049-34598	33695-34702	
CM11 + CM12	39573-40016	40189-39355	
CM15	40137-40506	40345-40626	
CM16	38041-37623	38000-37698	
CM17	52465-52147	52191-52514	
CM18	45227-45877	44818-45552	*lom*
CM19	45610-46100	45981-46382	
CM20	4098-3676	4333-4052	
CM21	39305-39919	39405-39650	
CM22	39875-40526	39909-40298	
CM23	45713-46232	a: 45784-45921	
		b: 46072-46239	

### Phage-encoded, lysogen-culture gene expression identified by 2D-PAGE

**Figure 2:**
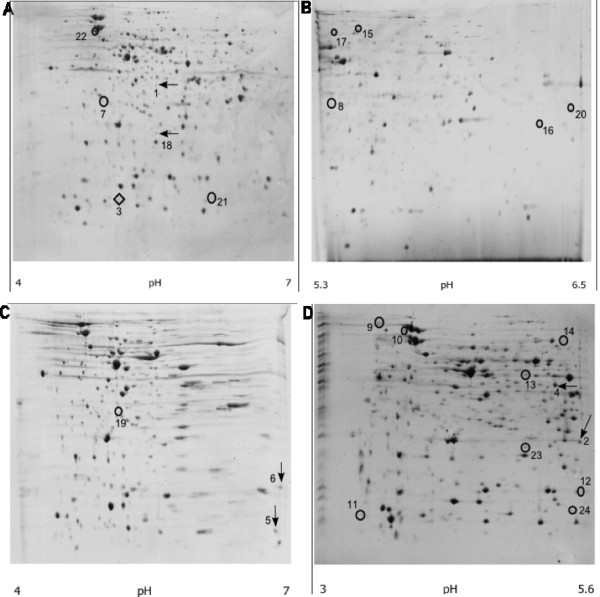
**2D-PAGE images of total cell protein from MC1061/Φ24_B_::Kan**. IEF on pH range 4-7 (A, C), 5.3-6.5 (B) and 3-5.6 (D). Arrows represent proteins identified as phage encoded; circles represent proteins identified as encoded by *E. coli*, but not present on corresponding naïve MC1061 gels (data not shown).

Reproducible sets of gels from 2D-PAGE analyses were obtained through the utilisation of IPG strips in the pH ranges of 3.5-5.6 and 5.3-6.5. The optimal protein concentration loaded on the gels was found to be 200 μg of total cellular protein from crude cell lysates. A total of 42 protein spots were found only in the lysogen gel sets (data not shown); these were excised from the gels and analysed by MALDI-TOF. Twenty-four of these spots (Figure 2) contained enough protein for the generation of mass spectral data. When these spectra were searched against the University of Liverpool MASCOT database, which included all of the Φ24_B _genome predicted proteins, six samples matched predicted phage proteins (P1 to P6, Table 2, Figure 1). The remaining 20 spots were identified as *E. coli *proteins (Table 2); these are potentially lysogen specific but were not investigated further here.

**Table 2:** Protein identities according to the MASCOT database

P♯	Gene name	**Access No**.	pI/MW (Da)	Description	Sequence^a ^Coverage (%)	MASCOT^b ^Score	Peptides^c ^matches	Estimated pI/MW (Da)	MASCOT Database Identified in
**1**	*P1*		5.28/33860	Identical to hypothetical protein p78 from 933 W^d^	32	63*	6	5.50/40000	1
**2**	*P2*		5.27/17096	Similar to hypothetical protein p23 from 933 W	42	39	5	5.00/15000	1
**3**	*P3*		5.09/13472	Similar to hypothetical protein p24 from 933 W	33	55	3	5.6/8000	1
**4**	*P4*		5.14/25800	Identical to exo of 933 W	29	52	5	5.5/40000	1
**5**	*P5*		5.29/7336	Not known homologue	83	41	2	7.00/7000	1
**6**	*P6*		5.22/13628	Identical to hypothetical protein Stx2Ip064^e^	38	30	4	7.00/10000	1
**7**	*nanA2*	Q6KD26	5.77/34077	N-acetylneuraminate lyse 2	21	47	4	5.3/35000	2
**8**	*gadB*	CAQ31981	5.29/52634	Glutamate decarboxylase beta	23	57	7	5.3/35000	2
**9**	*sodB*	P0AGD5	5.58/21121	Superoxide dismutase [Fe]	40	53	6	4.00/100000	2
**10**	*napA*	AAC75266	8.23/92983	Nitrate reductase	14	49	7	5.5/100000	2
**11**	*tig*	AAA62791	4.73/47994	Trigger factor	24	58*	7	3.5/6000	2
**12**	UTI89_C3021	Q1R837	4.70/6971	Hypothetical protein	70	42	2	5.5/7000	2
**13**	2FPKA	ZP_04873224	5.24/32497	6-phosphofructokinase	23	46	5	5.3/50000	2
**14**	*gcpE*	S23058	5.87/40658	1-hydroxy-2-methyl-2-(E)-butenyl 4-diphosphate synthase	16	38	4	5.5/80000	2
**15**	*aceE*	P0AFG9	5.46/99475	Pyruvate dehydrogenase E1 component	10	60*	7	5.4/100000	2
**16**	*bfpK*	Q9S141	7.63/18294	BfpK	54	49	3	6.4/25000	2
**17**	*ychN*	P0AB53	5.02/12685	ychN	46	38	2	5.3/100000	2
**18**	UTI89_C1147	Q1RDD	5.57/24945	Hypothetical protein	15	38	4	5.7/30000	2
**19**	*ompC*	Q9RH85	4.55/40474	Outer membrane protein	18	44	4	5.5/40000	2
**20**	ECs1247	G90784	4.74/25144	Hypothetical protein	26	39	5	6.5/35000	2
**21**	UTI89_C2748	Q1R8V6	10.19/10724	Hypothetical protein	44	50	4	6.4/8000	2
**22**	*nirB*	E86001	5.79/93112	Nitrate reductase (NAD(P)H) Subunit	10	53	8	5.3/100000	2
**23**	*yagP*	CAQ30761	5.65/15401	yagP protein	36	43	3	5.3/10000	2
**24**	*rhsF*	Q47284	5.69/23342	RhsF	18	42	4	5.69/8000	2

Table represents matches to *E. coli *proteins in the MASCOT database and matches to Φ24_B _proteins in the University of Liverpool local MASCOT database

^a ^percentage of sequence of the matched protein that is covered by the experimental MS.

^b ^logarithm of the probability that the match between the experimental data and a protein sequence in the database is a random event.

^c ^number of peptides that match the protein in the database

^d ^933 W is an Stx2 phage described by Plunkett *et al. *[16].

^e ^Stx2 is an Stx2 phage described by Sato *et al. *[20].

*represents significant matches (p-value < 0.05)

1 University of Liverpool local MASCOT database; 2 general MASCOT database

### Analyses of gene expression patterns

Generally, lambdoid phage regulatory circuits tightly control the expression of genes, yet some of the genes identified in the CMAT library and the 2D-PAGE analyses above were phage genes whose expression should be linked to prophage induction (Figure 1) and not the stable prophage state, *e.g*. the gene encoding the tail spike protein. It was assumed that gene expression normally linked to the lytic replication cycle must be at a very high level in a small subset of the cells and that lysogen-restricted gene expression patterns of these genes might be very low, especially as neither CMAT nor 2D-PAGE identified the expression of repressor, the product of the *cI *gene, in the lysogen culture. Therefore it was essential to devise a method that would determine whether phage genes were being expressed by the majority of the stable lysogen population, or the small subset of the population undergoing spontaneous induction events. A strategy involving qPCR was developed to provide this important information, and a variety of genes were chosen as controls for this study (Additional file 1: Table S1, Figure 1). Calibration curves for quantitation and comparison of the qRT-PCR data were produced for every set of primers used; R^2 ^values from linear regression analyses of these standards ranged from 0.990 to 0.999 with slopes ranging between -3.72 and -3.10 (Additional file 1: Table S1).

**Figure 3:**
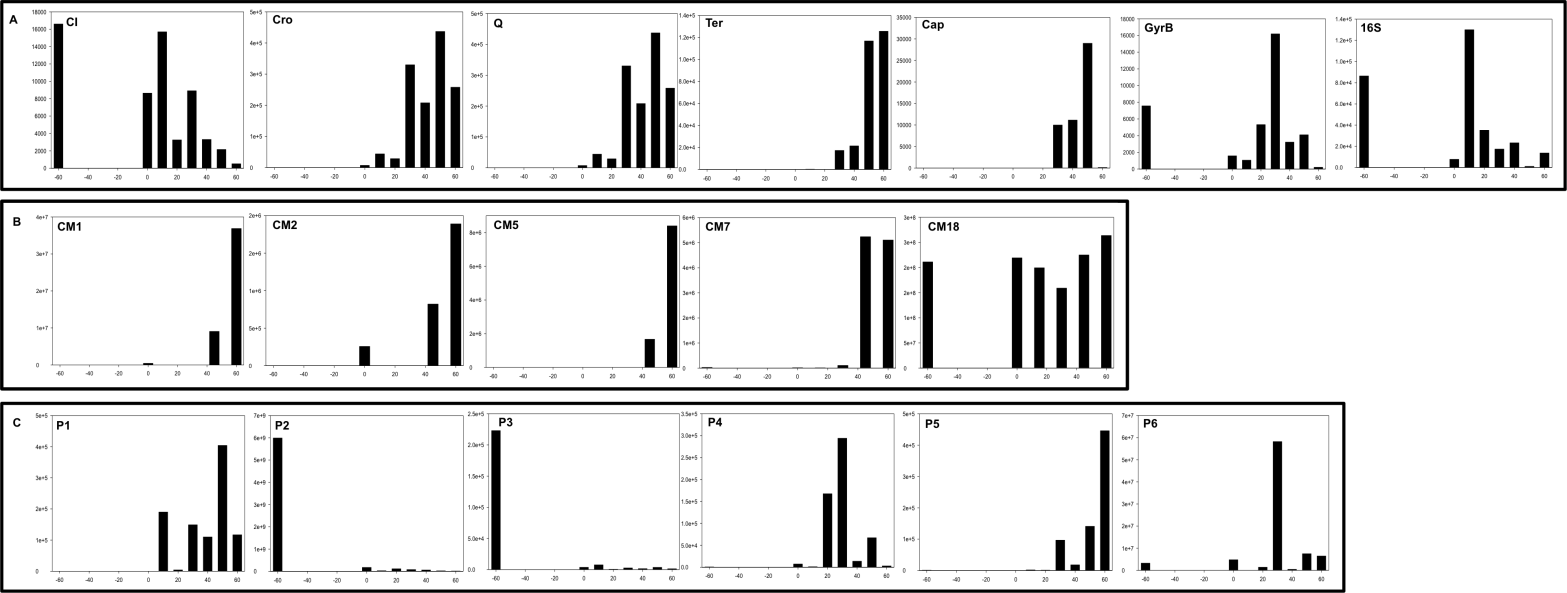
**Graph depicting gene expression profiles before and following norfloxacin induction**. Panel A: Control Genes. CI, marker gene for lysogeny-restricted expression; **Cro and Q**, marker genes for early induction response; **Ter and Cap**, marker genes for late gene expression; **GyrB and 16S**, marker genes for the cellular response. **Panel B: **Expression profiles of the prophage genes identified by CMAT. **Panel C: **Expression profiles of the genes identified by 2D-PAGE analyses of the lysogen. The Y axis represents gene copy number per 300 ng of RNA; the X axis represents time (min). Time -60 refers to the samples taken before induction and represents the lysogen population, Time 0 represents samples taken at the beginning of the recovery time, Time 10, 10 min after recovery, *etc*. The experiment was run using biological replicates, but due to the asynchronicity of induction across these experiments the data from a representative single biological replicate are shown.

The data from the qPCR assay were analysed by comparing the shape of the expression data for any given gene from a lysogen culture throughout the prophage induction process where time 0 is the point of norfloxacin (inducer) addition (Figure 3). Lysogen-restricted gene expression should be negatively affected after induction (Figure 3A, CI), and if expression is actually linked to the small proportion of cells undergoing spontaneous induction, then the expression levels should rise during the induction process. This is indeed the case as expected for Q, Cro, Capsid & Terminase, which display a significant increase after 50 min of recovery, Figure 3; Additional file 2: Table S2).

Four genes identified by 2D-PAGE, P1, P4, P5 and P6, visibly follow the same expression pattern as the genes expressed during the lytic cycle and accordingly the increase in gene copy number is significant (p-value < 0.05) after 50 or 60 min of recovery from exposure to norfloxacin (Figure 3; Additional file 2: Table S2). P3 and P2 appear to have a similar pattern to *cI*, i.e. their levels of expression in the lysogen are higher than the levels after induction; however the ANOVA analysis did not identify these differences as significant, probably due to the high variability amongst the replicates. Of the five CDS identified by CMAT, which were subsequently selected for expression analysis based upon either their putative function or location within the phage genome, four had expression patterns linked to the lytic cycle. CM18 was shown by qPCR to be strongly expressed in lysogen cultures, but when the cells are induced, high expression levels are maintained, suggesting that expression of this gene has been uncoupled from the phage regulatory circuits. The outcome of one-way ANOVA analysis to determine the impact of prophage induction on gene expression was found to be significant in 11 cases (p-value < 0.05): cI, cro, terminase, capsid, Q, CM1, CM2, CM5, CM7, P1 and P5. The other 7 genes studied did not present significant changes in expression: P2, P3, P4, P6, CM18, 16S, and gyraseB. The full set of p-values for the data in Figure 3 are presented in Additional file 2: Table S2.

## Discussion

Temperate phages, maintained as prophages in their lysogens, have been the subject of speculation concerning their benefit to the host: selective advantage, increased virulence, and other traits with varying degrees of direct and/or indirect impact on the host have been identified [11,21-27]. The challenge in this area has been how to identify phage-encoded genes that directly affect their lysogen, because many/most phage genes are annotated as encoding hypothetical proteins. In addition, there will always be a small background population undergoing spontaneous induction in the absence of discernible stimuli [19], potentially confounding the identification of lysogen-restricted prophage gene expression. In a specific *E. coli *lysogen of Stx2-phage 933W, a phage very closely related to Φ24_B_, the spontaneous induction rate was calculated as 0.014% [28], which means that in a lysogen culture fourteen cells per 100,000 are undergoing prophage induction. Other recent work was demonstrated that various induction agents and growth conditions differentially effects induction in a prophage-dependent manner [29]. Assuming a burst size similar to that of bacteriophage Lambda (170 ± 10 virions cell^-1^) [27], a significant amount of phage structural protein production can occur in an uninduced lysogen culture.

In order to mitigate this effect, the growth phase at which the ratio of lysogens to free phage was high (two to three hours post inoculation) was targeted. However, the cell density at this point was very low and 5-6 hours was chosen as the standardised incubation time as a compromise. In this study, 26 genes from the bacteriophage Φ24_B _were identified by either CMAT or 2D-PAGE as being expressed in *E. coli *lysogen culture. No genes were identified by both CMAT and 2D-PAGE methods, perhaps due in part to the low absolute number of Φ24_B _genes identified by the latter approach. However, the level of redundancy in the genes identified by the CMAT clones was lower than expected, given the number of clones screened and the calculated phage genome coverage; however, putative positive clones were selected conservatively in an attempt to limit the number of false positives. Additionally, CMAT-based identification may also introduce bias into library screening due to differences in protein immunogenicity and antigenicity. It is important to note that the best characterised lysogen-restricted gene, *cI *(encoding lambdoid phage repressor), was not identified using either CMAT or 2D-PAGE, indicating that this study was not exhaustive. Nevertheless, the paucity of information on lysogen-restricted gene expression is such that these data represent a significant step forward in our understanding of phage/host interactions and lysogen biology.

Of the 26 phage genes identified in this study, Tsp, encoding the characterised tail spike protein of Φ24_B _[30,31] was a known structural protein and therefore not expected to be expressed by a stable lysogen (Tables 1 &3), while the expression profiles of the other 25 proteins were unknown. Therefore the resulting challenge was to identify the fraction of the culture (lysogens or cells undergoing lysis) that were responsible for expression of these 26 phage genes as well as determining testable hypotheses to assign function to the identified gene products. Five genes identified during the CMAT screening were chosen for gene expression profiling due to their genome location, potential function or degree of conservation across a range of phages (Table 3). The CDS CM18 encodes a Lom orthologue, which was expected to be expressed in the lysogen as the lambda *lom *gene is associated with the alteration of the lysogen's pathogenic profile after location of Lom in the outer membrane [32-34]. However, expression of *lom *in the Φ24_B _lysogen unexpectedly appears to be uncoupled from the phage regulatory pathways, because it is expressed at similar levels in an infected cell regardless of whether that cell exists as a stable lysogen or is undergoing prophage induction. The CDS CM2 encodes a putative Dam methyltransferase. Bacterial-encoded Dam methyltransferase has been shown to be essential for maintenance of lysogeny in *E. coli *infected with Stx-phage 933 W [35]. The expression pattern of the Φ24_B_-encoded Dam methyltransferase could indicate that it is fulfilling a similar role, or supplementing the function of the host-encoded Dam methylase in lysogens infected with this phage. The functions of CM5 and CM7 are unknown. CM7 is an ORF of 8 kb, and as the amount of DNA that can be packaged by a phage is limited, such a large gene is likely to be conserved only if it confers an advantage to the phage or its lysogen; it may be significant that this large gene is associated with several other phages (Table 3). CM5 is a small CDS located on the complementary strand to the one encoding CM7, in a region with few other CDS, though it is directly upstream of another CMAT-identified CDS, CM6. The data (Figure 3) indicate that the expression of these 3 genes is linked to prophage induction, a surprising outcome as CM7 does not appear to be a phage structural gene, has been indicated by bioinformatic analyses (data not shown) to be a probable outer membrane protein, and is downstream of CM18, whose regulation is uncoupled from expression of the late genes.

**Table 3:** Distribution of the proteins identified by CMAT and 2D-PAGE across phage genomes

Gene	Other Stx phages carrying the proteins in the study (identity)	Accession number	Other phages	Accession number
CM1	Stx2 converting phage II (99%)	YP_003828920.1		
	phage VT2-Sakai (99%)	NP_050557.1		
	phage 933 W (99%)	NP_049519.1		
	Stx1 converting phage (99%)	YP_003848832		
	phage BP-933 W (99%)	YP_003848832.1		
	phage VT2phi_272 (99%)	ADU03741.1		
	phage Min27(100%)	ADU03741		

CM2	Stx2 converting phage II (100%)	BAC78116		
	phage VT2-Sakai (100%)	NP_050531.1		
	phage Min27 (100%)	YP_001648926		
	phage HK97 (99%)	AAF31137		
	phage Lahn2 (99%)	CAJ26400		
	phage Lahn3 (98%)	CAC95062.1		
	phage 2851 (99%)	CAQ82016		
	phage CP-1639(99%)	CAC83142		
	prophage CP-933 V(99%)	AAG57233		
	Phage Nil2 (99%)(99%)	CAC95095		
	Stx1 converting phage (99%)	YP_003848889.1		
	Phage CP-1639 (99%)	CAC83142.1		
	Phage YYZ-2088 (99%)	YP_002274170.1		
	Stx2-converting phage 1717 (99%)	YP_002274244.1		

CM5	phage Min27 (100%)	YP_001648966.1		
	Stx2 converting phage II(100%)	YP_00388933.1		
	Stx2 converting phage I(100%)	NP_612929.1		
	phage VT2-Sakai (100%)	NP_050570.1		
	phage 933 W (100%)	NP_049532.1		
	phage VT2phi_272 (100%)	ADU03756		

CM7	phage VT2-Sakai (99%)	NP_050570		
	Stx1 converting phage (99%)	BAC77866.1		
	Phage VT2phi_272 (97%)	ADU03756.1		
	Phage 933 W (97%)	NP_049532.1		
	Stx2 converting phage I (97%)	NP_612929.1		
	Stx2 converting phage II(97%)	BAC78032.1		
	Phage BP-933 W (97%)	AAG55616.1		
	Stx2 converting phage 86 (91%)	YP_794082.1		
	Phage Min27 (97%)	YP_001648966.1		

CM18	phage VT2-Sakai (100%)	NP_050564.1		
	Stx1 converting phage (100%)	YP_003848839.1		
	Phage 933 W (100%)	NP_049526.1		
	Stx2 converting phage I (100%)	ZP_02785836.1		
	Stx2 converting phage II (100%)	YP_003828926.1		
	Phage BP-933 W (100%)	NP_286999.1		
	Stx2 converting phage 86 (97%)	YP_794076.1		
	Phage Min27 (100%)	YP_001648959.1		

P1	Stx2 converting phage II (99%)	YP_003828937.1	Phage phiV10 (78%)	YP_512303.1
	Stx2 converting phage I (99%)	NP_612952.1		
	Phage 933 W (99%)	NP_049538.1		
	Phage BP-933 W (99%)	AAG55619.1		
	phage VT2-Sakai (99%)	NP_050575.1		
	Phage Min27 (96%)	YP_001648901.1		
	Stx2-converting phage 86 (96%)	YP_794094.1		
	Phage BP-4795 (96%)	YP_001449244.1		
	phage CP-1639 (74%)	CAC83133.1		

P2	Stx2 converting phage I (100%)	NP_612997.1	*Salmonella enteric*	YP_002455860.1
	Phage 933 W (100%)	NP_049484.1	bacteriophage SE1 (86%)	
	Phage BP-933 W (100%)	AAG55573.1	*Salmonella *phage ST160 (86%)	YP_004123782.1
	Phage Min27 (100%)	ABY49878.1		
	Stx2-converting phage 86 (100%)	YP_794109.1		

P3	Stx2 converting phage I (100%)	NP_612995.1		
	Phage 933 W (100%)	NP_049483.1		
	Stx2-converting phage 86 (100%)	YP_794108.1		
	Phage Min27 (100%)	YP_001648915.1		
	Phage BP-933 W (100%)	AAG55572.1		

P4	Phage 933 W (100%)	NP_049473.1	Phage lambda (98%)	NP_040616.1
	Phage BP-933 W (100%)	NP_286952.1		
	Prophage CP-933 V (100%)	NP_288695.1		
	Stx2 converting phage I (100%)	NP_612980.1		
	Phage VT1-Sakai (100%)	BAB19617.1		
	Phage YYZ-2008 (99%)	YP_002274150.1		
	Stx2-converting phage 1717 (98%)	YP_002274221.1		
	prophage CP-933 K (98%)	YP_003500773.1		
	phage BP-4795 (98%)	YP_001449249.1		
	phage Min27 (99%)	YP_001648905.1		

P5	Stx2 converting phage I (100%)	NP_613032.1		
	Phage 933 W (100%)	NP_049503.1		
	Stx2 converting phage II (100%)	BAC78139.1		
	Stx2-converting phage 1717 (98%)	YP_002274255.1		
	phage 2851 (98%)	CAE53952.1		
	Phage BP-4795 (97%)	YP_0014419282.1		

P6	Stx2 converting phage I (99%)	NP_612943.1		
	Stx2 converting phage II (99%)	BAC78046.1		
	phage VT2phi_272 (99%)	ADU03756.1		
	phage Min27 (99%)	YP_001648966.1		
	phage VT2-Sakai (99%)	NP_050570.1		
	Stx1 converting phage (99%)	BAC77866.1		
	Stx2-converting phage 86 (96%)	BAF34067.1		

The qPCR expression profile for the phage genes identified as being expressed in the lysogen by 2D-PAGE, P1, P2, P3, P4, P5 and P6, indicated that only the expression of P2 and P3 were restricted to lysogen cultures with a stable prophage. The genes for both P2 and P3 lie downstream of the *cI *gene. However, their expression levels are one and five orders of magnitude greater, respectively, than the expression levels of *cI*, the lambdoid phage repressor gene. It is known that in Lambda phage, the *cI *gene transcript is leaderless, possessing no ribosome binding site for initiation of translation, with transcription and translation beginning at the AUG start codon [36]. If this causes the 5' end of the transcript to be less stable and more easily subject to degradation, the higher level of P3 transcript could simply be due to possession of a longer half life than those genes at the 5' end of the transcript.

The genes encoding P2 and P3 are conserved in many other phages (Table 3). They have no bioinformatically identifiable promoters of their own, so are likely to be driven by *pRM *or *pRE *like *cI *(see [37] for a cogent review of the related lambda phage), but differences in the levels of transcription between these 3 genes implies that there is still more to discover about the right operator region of this phage. The proteins P1, P4, P5 and P6 all exhibit gene expression profiles that suggest they are expressed following prophage induction. These genes are scattered across the phage genome (Figure 1) and are shared by various phages (Table 3). The protein P4 appears to be part of the lambda Red recombinase system [38-40] and the data presented here suggest that this is most active upon prophage induction. This could be relevant to the mechanisms that underpin diversification, evolution and production of new phages by lysogens carrying an inducible prophage along with one or more inducible or remnant prophages [11,41,42]. The proteins P1, P5 and P6 are scattered across the genome on the strand typically associated with expression of genes linked to lysogenic infection (*e.g. cIII, N, cI*). Two genes encoding proteins P1, P5 and P6 are found in other phages, but have no known function.

In summary, genome sequencing of prophages and bacteriophages has identified that these viral elements encode higher numbers of hypothetical genes than those to which we can currently assign a function. These genes are often conserved across many bacteriophages, but do not appear to encode structural proteins. For these genes to remain present in the phage genome, especially considering the fluidity of the genetic composition of lambdoid phages [43], they must surely provide an important function in either the phage life cycle or that of the lysogen itself. In attempting to identify prophage genes whose expression was restricted to the stable prophage state, our goal was to identify prophage genes that were candidates for influencing the fitness of the bacterial host. However, the study was hampered by the fact that lysogen-restricted gene expression can be at very low levels, and phage genes associated with phage replication are expressed at very high levels.

## Conclusions

Two different experimental strategies were employed to identify prophage genes expressed by their lysogen, and it is interesting to note that lysogen-specific antibody recognition of a peptide expression library and differential 2D-PAGE with subsequent protein identification by peptide mass spectrometry, did not identify the same genes or proteins. The failure of both to identify expression of the *cI *gene encoding the phage repressor was shown by RT-qPCR to be due to the very low expression levels peculiar to this phage gene (Figure 4); the CI protein is also very susceptible to autocatalysis and therefore elusive. Both CMAT and 2D PAGE identified some phage genes that were associated with lytic induction, and the qPCR strategy was useful for discriminating low level expression in stable lysogens from high-level gene expression in the minority of lysogens that were undergoing spontaneous induction. Improving our understanding of the STEC disease process is ever more urgent in light of the recent emergence of a new Shiga-toxin producing *E. coli *pathotype [44], and determining the function and expression patterns of the genes in Stx phage genomes is very important in that context.

## Methods

### Bacterial strains and culture

The *E. coli *K-12 strain, MC1061, was used as the bacterial host for the production of lysogens. MC1061(Φ24_B_) refers to the Φ24_B _lysogen of MC1061; naïve MC1061 refers to cells that have not been infected by Φ24_B_. *E. coli *K-12 strain DM1187 was used as the indicator host strain in plaque assay experiments [18]. BL21-AI cells (Invitrogen, Paisley, U.K.) were used as the expression host for genetic constructs. Bacterial strains, plasmids and phages used in this study are listed in Table 4.

**Table 4:** Bacterial strains, plasmid and phages used in the study

*E.coli *strains, plasmids and phages	Relevant Genotype	Reference
**BL21-AI**	F- *ompT hsdSB*(rB-, mB-) *gal dcm *(DE3), arabinose inducible T7 RNA polymerase	Invitrogen, Paisley, U.K.
**MC1061**	F^- ^Δ(*ara*-*leu*)7697 Δ(*codB*-*lacI*)3 *galK16 *λ^- ^*mcrA0 rpsL*150(strR) *mcrB1*	[18]
**DM1187**	F- *dam*-13::Tn9(Cm^R^) *dcm*- *mcrB hsdR*-M + *gal1 ara*- *lac*- *thr*- *leu*- *tsxR*	[45]
**TOP10**	F- *mcrA *Φ80*lacZ*ΔM15 *recA*^+^	Invitrogen, Paisley, U.K.
**pCR^®^-Blunt**	*lacZ *α, Kan^R^, *ccdB*	Invitrogen, Paisley, U.K.
**pET30c**	Expression vector with T7 promoter, Kan^R^, Tet^R^,	Novagen, Notts, UK
**Φ24_B_**	Stx2-phage, Δ*stxA*_2_::*aph3*	[14]

All cultures, unless otherwise stated, were propagated from an overnight (~16 h) starter culture (0.5% v/v inoculum) in Luria Bertani (LB) broth (Merck KGaA, Darmstadt, Germany) containing 0.01 M CaCl_2_, incubated at 37°C with shaking at 200 r.p.m. Lysogen cultures were grown in the presence of kanamycin (Kan, 50 μg ml^-1^). Induction of protein expression in BL21-AI cells took place in BHI broth with 0.2% arabinose and 1 mM IPTG.

### Induction of phage lysogens

Cultures of MC1061(Φ24_B_) cells were incubated with norfloxacin (1 μg mL^-1^) for 1 h at 37°C with shaking at 200 r.p.m. Cultures were then diluted 1:10 in fresh LB and the bacteria allowed to recover from the growth inhibitory effects of the antibiotic for 1 h at 37°C (the recovery period), with shaking at 200 r.p.m.

### Antisera production for use in CMAT

A 2 L culture of MC1061(Φ24B) was propagated for 6 hours. The cells were pelleted and resuspended in 1 ml of retained supernatant plus 1 ml of LB broth. Protease inhibitors (20 μL) (Roche Complete Mini EDTA Free protease inhibitor cocktail tablets, Bath, U.K.) and 10 μL of lysis buffer (7 M urea, 2 M thiourea, 2% CHAPS, 1% DTT, Roche Complete Mini EDTA-free protease inhibitor cocktail tablets) were added to each. The samples were sonicated at 15-18 μ for 6 × 10 s bursts. Absolute methanol (1.5 ml) was added, and the samples were incubated at -20°C for 60 min. Protein was harvested by centrifugation at 16,000 *g *for 5 min, and the resultant protein pellets were air-dried and suspended in 0.5 ml phosphate buffered saline (PBS). The samples were pooled; the protein content was measured by Bradford Assay [46] and adjusted to 1 mg ml^-1^. A total of 4 mg of the lysogen protein was sent to Eurogentec (Seraing, Belgium) for antisera production in rabbits, using the Ribi adjuvant system. Two rabbits were immunised with the protein sample on days 0, 14, 28 and 56 of the program. Bleeds were carried out on days 0 (pre-immune sera), 38, 66 and 87 (final bleed). Pre-immune sera from the two rabbits used were received and tested for cross-reactivity by western blot analysis.

CMAT was carried out as per instructions from the license holder, Oragenics Inc., FL., U.S.A. [17,47], with the exception that BL21-AI was used as the expression strain for the phage library. The recommended expression host, BL21[DE3], is an *E. coli*-λ lysogen, and therefore an inappropriate strain to use in phage protein expression studies [48]. The expression library was created from Φ24_B_::Kan DNA. The rabbit antisera were depleted of antibodies reactive to *E. coli *proteins by a series of adsorptions to naïve MC1061 whole cells and cellular lysate, and to BL21-AI + pET30c (empty vector) whole cells and cellular lysate. The depleted antisera were compared to undepleted antisera by western blot. Adsorptions were repeated until no bands were detectable by western blot probing of 6 μg of naïve MC1061 proteins.

### Peptide expression library construction

Semi-confluent plaque assay plates [18] were overlaid with 3 ml SM buffer (100 mM NaCl, 8 mM MgSO4, 50 mM Tris-HCl, pH 7.5) and incubated at 4°C for 16 h, with gentle agitation. The SM buffer and top agar were transferred to separate 50 ml centrifuge tubes that were vortexed with 10% (v/v) fresh SM buffer and subjected to centrifugation at 10,000 *g *for 10 min. The supernatant was pooled and 30 μl of chloroform were added to each 10 ml of buffer. DNase (5 μg ml^-1^) and RNase (1 mg ml^-1^) were added, and the samples were incubated at 37°C for 1 h. PEG 8000 (33% [w/v]) was added, and the samples were incubated on ice for 30 min. Precipitated phage particles were harvested by centrifugation for 10 min at 10,000 *g*, and the pellets were resuspended in 500 μl SM buffer per 30 ml starting volume. Samples were treated with DNase and RNase, as before. Phage DNA was purified by phenol:chloroform:isoamyl alcohol extraction and isopropanol precipitation [49] and resuspended in 100 μl ddH_2_O. The Φ24_B _DNA (15 μg ml^-1 ^in TE) was fragmented using a HydroShear (GeneMachines, MI, USA), at speed code 6 for 30 cycles, followed by 30 cycles at speed code 2. DNA of the required size range (300-900 bp) was isolated by gel purification. pET30c plasmid (EMD Biosciences) DNA was digested with *Eco*R V and dephosphorylated with calf intestinal phosphatase (New England Biolabs) according to the manufacturer's recommendations. The size fractionated Φ24_B _DNA fragments were cloned into the prepared pET30c DNA (50 ng) vector in a molar ratio of 25:1 (insert to vector). Chemically competent BL21-AI expression host cells (Invitrogen) were transformed with the plasmid DNA according to the manufacturer's recommendations.

### Primary screening

Transformed BL21-AI cells were plated onto LBKan plates and incubated at 37°C (11 h). Nitrocellulose membrane (0.2 μm pore size, BioTraceTM) was laid onto the top of each plate for approximately 1 min. The membranes were transferred colony-side up to LBKan agar plates supplemented with arabinose (0.2%) and IPTG (1 mM), and incubated at 37°C for 3 h. The master plates were incubated for a further 3 - 5 h at 37°C, until the colonies reached a diameter of 1-2 mm. The membranes were lifted from the agar plates and placed on chloroform-saturated filter paper, colony-side down, for 1 min, after which the chloroform was allowed to evaporate completely. The membranes were then gently agitated in blocking solution (PBS plus 0.5% Tween 20 and 5% skimmed milk powder) for 1 h at ambient temperature, washed in PBST (PBS plus 0.5% Tween 20; 4 × 10 min) with gentle agitation and probed with the primary antibodies (depleted antisera) in 10 ml PBST (1:1,250) for 16 h at 4°C with gentle agitation. The membranes were then washed four times in PBST and agitated for 1.5 h in secondary antibody solution (HRP conjugated to goat anti-rabbit IgG [Sigma]) (1:30,000). The membranes were washed four times in PBST, rinsed twice in PBS and washed for 10 min in PBS, under gentle agitation. Enhanced chemiluminescent (ECL) reagent was used to develop the membranes and the chemiluminescence was visualised by exposure of Roche Lumi-Film Chemiluminescent Detection Film to the membranes. Putative positive clones were identified on the master plates and each one was transferred to fresh LBKan agar.

### PCR verification of insert

For verification of the presence of cloned DNA, putative positive colonies were used as the template source for a colony PCR and the T7 promoter and T7 terminator primers (Novagen, Notts, U.K.). Thermal cycling conditions using Taq polymerase comprised an initial denaturation of 5 min at 94°C, 35 cycles of denaturation at 94°C for 30 s, annealing at 55°C for 30 s, extension at 72°C for 30 s kb^-1 ^product, followed by a final extension at 72°C for 7 min.

### Secondary screening

Putative positive colonies were cultured overnight in BHI Kan (1 ml), at 37°C, without shaking. The cells were harvested by centrifugation at 16,000 *g*. The supernatant was decanted and the cells resuspended in 20 l BHI Kan. Each suspension was spotted in triplicate (1 μl) onto duplicate nitrocellulose membranes and placed on a BHI Kan agar plate. The plates and membranes were incubated for 3 h at 37°C, the membranes removed and one of the duplicate membranes overlaid onto a LB Kan agar plate supplemented with 0.2% arabinose and 1 mM IPTG while the other membrane was placed onto a LB Kan agar plate. These were incubated for 3 h at 37°C. The membranes were removed from the plates, and placed on chloroform- saturated filter paper for 1 min. Once dry, 1 μl of the lysogen-specific antiserum was spotted onto the bottom of the membrane, as a positive control. Antibody reactivity was determined as described above for primary screening.

### DNA sequencing

Plasmid DNA was sequenced by GATC Biotech (Konstanz, Germany), using the T7 promoter and terminator primers. Sequences were translated using ExPASY's *Translate *tool http://www.expasy.ch/tools/#proteome. The sequences were aligned to the annotated Φ24_B _genome [GenBank:HM_208303] and CDS in-frame with the expression vector were documented.

### qPCR

Induction of MC1061(Φ24_B_) cultures was performed as described above. A 1 ml sample was taken before addition of norfloxacin to the cultures, and further 1 ml aliquots removed at 10-15 min intervals throughout the 60 min recovery time. RNA was immediately extracted and DNAse treated with TURBO™ DNAse (Ambion, TX, USA) according to the manufacturer's instructions. Absence of DNA was verified by qPCR. Each RNA sample (300 ng) was reverse transcribed using random hexamer oligonucleotides (Bioline, London, UK). Specific primers were designed to amplify an approximately 100 bp region of each gene in the study (Additional file 1: Table S1). qPCR was performed using a StepOnePlus™ Real-Time PCR System (Applied Biosystems); each reaction consisted of 1 μl of cDNA, 1 x SensiMix*Plus *SYBR (Quantace, London, U.K.), 200 nM of specific primers in a 25 μl reaction. The amplification cycling conditions were: initial denaturation at 95°C for 10 min; 39 cycles of denaturation at 95°C for 10 s; annealing at 60°C for 30 s; extension at 72°C for 5 s. A melting curve analysis was performed for each amplification reaction, with a temperature gradient of 0.1°C from 55°C to 95°C. No-template controls and a calibration curve, consisting of 6 dilutions of the PCR amplicon of each gene cloned into PCR-Blunt vector (Invitrogen, Paisley, U.K.) linearised with *Nco *I (NEB, Herts, U.K.), were included in every experiment (Additional file 1: Table S1). Statistical analysis was performed using a one-way ANOVA comparing gene copy numbers at different time points in each experiment to test the hypothesis that there is no variation in gene copy number during the recovery period. A *post-hoc *Dunnett's test was employed, using the sample corresponding to the lysogen culture (-60) as the reference group, to assess whether or not time points differed from the reference. P < 0.05 values were considered to be statistically significant.

### Protein extraction for 2D-PAGE

Cultures of MC1061 and MC1061(Φ24B) were incubated for 6 h at 37°C. Cells were harvested and pellets washed in 1 ml of wash solution (10 mM Tris-HCl, pH 8.0; 1.5 mM KH_2_PO_4_; 68 mM NaCl; 9 mM NaH2PO4). Cells were resuspended in 1 ml of resuspension buffer (10 mM Tris-HCl, pH 8.0; 1.5 mM MgCl2; 10 mM KCl; 0.5 mM DTT; 0.1% SDS; 20 μl of protease inhibitor [Roche CompleteMini EDTA Free protease inhibitor cocktail tablets]) and each sample was sonicated for 5 × 10 s. DNase was added (5 μg ml^-1^) and samples were incubated for 1 h at 37°C. Samples were centrifuged for 1 h at 12,000 g, the supernatant recovered and protein concentration determined using the Bradford Assay. Aliquots (110 μg protein) of the sample were taken and precipitated in 10% TCA in acetone containing 20 mM DTT for 45 minutes at -20°C. Pellets were washed twice in ether.

### 2D-PAGE

Isoelectric focussing was carried out on 18 cm IPG strips (pH 4-7,3-5.6 and 5.3-6.5;GE Healthcare), at 3,500 V for 7 h. Proteins were separated in the second dimension on 1.5 mm 4% stacking/15% resolving SDS-PAGE gels, for 6.5 h at 20 W per gel (up to maximum of 180 W). Proteins were silver stained [50].

### In-gel digestion of protein samples

This was carried out according to the protocol described by Courchesne & Patterson [51] with the following modifications: protein spots were excised from the gel and destained with 50 μl of destaining solution (30 mM potassium ferricyanide, 100 mM sodium thiosulphate) until the silver stain disappeared; protein digestion proceeded in 25 mM ammonium carbonate/trypsin (5 ng μl^-1^) at 37°C for 16 h.

### Matrix-assisted laser desorption/ionisation-time-of-flight (MALDI-TOF) mass spectrometry

Trypsin-digested protein samples were added to an alpha-cyano 4-hydroxycinnamic acid matrix (LaserBioLabs, France) at a concentration of 10 mg ml^-1 ^in 50% ethanol: 50% acetonitrile: 0.1% TFA. Samples were analysed by MALDI-TOF on an ABI Voyager DE Pro (MALDI-TOF). The mass spectra generated were processed using Data Explorer to clean the spectra and isolate monoisotopic peaks (all Applied Biosystems). The Mascot Peptide Mass Fingerprint Database was used to search for homologues.

## Authors' contributions

LMR carried out the CMAT analyses and determined the growth and sampling times for the lysogen cultures. MV-G carried out the 2D-PAGE analyses, developed and performed the qRT-PCR assays and produced the figures. MH prepared all DNA samples for CMAT library production. JDH and MH designed CMAT and were involved in technical critiquing of these experiments. AJM and HEA designed the study and were involved in the interpretation of all data. All authors were involved in the writing and editing of this manuscript including the reading and approval of the final version.

## Supplementary Material

Additional file 1 **Table S1**. PCR amplification primers used in this study. A compilation of all of the amplification primers used in this study along with amplification efficiency information. Click here for file

Additional file 2 **Table S2**. Significance of Dunnett's test results for gene expression data in Figure 3: Results of the Dunnett's test to determine significance of gene expression profile differences before and after prophage induction. Click here for file
