# Supplementary material for: Identification of genes expressed in cultures of E. coli lysogens carrying the Shiga toxin-encoding prophage Φ24B
Source: BMC Microbiol. 2012 Mar 22;12:42. doi: 10.1186/1471-2180-12-42 (PMC3342100; doi:10.1186/1471-2180-12-42)
Supplement: Additional file 1 — Table S1. PCR amplification primers used in this study. A compilation of all of the amplification primers used in this study along with amplification efficiency information. [file 1471-2180-12-42-S1.DOC]

**Supplementary Table 1 PCR amplification primers used in this study**

| **Primer** | **Sequence (5' – 3')** | **Dilution range in gene copy number** | **R^2^** | **Slope** | **Proposed**  **function** |
| --- | --- | --- | --- | --- | --- |
| Term qPCR F | CGGTTTGTTCATTGCCTTCTCAACCG | 5.08 x10^5^ - 2540 | 0.990 | -3.22 | Cleaves concatemeric phage DNA, expressed during the lytic cycle |
| Term qPCR R | CCTAAATACAGCGCCAGAGTGC |
| Q qPCR F | GTAAAATCACGTCCACAGTGC | 4.47 x10^5^ - 1830 | 0.992 | -3.27 | Antiterminator, expressed during the lytic cycle |
| Q qPCR R | AACACGTAATAATCAACCAGC |
| Cro qPCR F | AAAGGGCTGTCTATAAGTGG | 7.61x10^5^ - 1520 | 0.992 | -3.07 | Transcription repressor, expressed during the lytic cycle |
| Cro qPCR R | GCCACCAGAAATCTCTTCG |
| Capsid qPCR F | AGGTGCCTGCGAAGCTATTC | 4.47 x10^5^ - 1830 | 0.991 | -3.10 | Structural gene, Antiterminator, expressed during the lytic cycle |
| Capsid qPCR R | GCTCTCCTGGTCACGACG |
| cI  qPCR F | gtgagggaacggagctacag | 3.55 x10^6^ - 1550 | 0.998 | -3.17 | Transcription repressor necessary to establish lysogeny, expressed in the lysogen |
| cI  qPCR R | gcggccttatgctttcaatg |
| 16s qPCR F | CATCGAGGAACGGTACGAGA | 6.35 x10^5^ - 1270 | 0.995 | -3.66 | Cell marker |
| 16s qPCR R | CGATCTCGGTAAAGTCGTCGAT |
| GyrB qPCR F | gtcgaagtggcgttgcagtg | 7.12 x10^5^ - 4060 | 0.996 | -3.12 | Target of norfloxacin, used as induction marker |
| GyrB qPCR R | agcctgccaggtgagtaccg |
| P1 qPCR F | cagcgtttgcataagcc | 3.55x10^5^ - 1730 | 0.997 | -3.21 | Phage gene of unknown function |
| P1 qPCR R | CGTGAAAAGGCAGAGAAAGC |
| P2 qPCR F | Cggataccatgcggacg | 3.55 x10^5^ - 1730 | 0.996 | -3.68 | Putative RuvC resolvase |
| P2 qPCR R | Cgttttgccgttctttttggtgg |
| P3 qPCR F | Gcggtgtgacttcaatatttc | 5.51 x10^5^ - 2360 | 0.990 | -3.50 | Phage gene of unknown function |
| P3 qPCR R | Gctgccatacgcgttactgaatc |
| P4 qPCR F | ggattcagtaacattcacgccg | 5.51 x10^5^ - 2360 | 0.991 | -3.25 | Putative lambda like exonuclease |
| P4 qPCR R | gcaaaaccccgatcaggaaagaag |
| P5 qPCR F | GCAGAGAGCGGTGAAGTTCAGC | 4.47 x10^5^ - 1830 | 0.998 | -3.28 | Phage gene of unknown function |
| P5 qPCR R | CGTCTCCGTCACTTCCTGCAG |
| P6 qPCR F | GCAAAACGGCAAGAAAAACCACC | 5.51 x10^5^ - 2360 | 0.998 | -3.36 | Phage gene of unknown function |
| P6 qPCR R | GCCTATGGTACGCCTGC |
| CM1 qPCR F | GAAATCTCCTGATGGTGAGG | 3.55 x10^5^ - 1730 | 0.996 | -3.70 | Tail spike protein |
| CM1 qPCR R | GATCCATCGTCATTCC |
| CM2 qPCR F | AAGGACTGCTGGCAAACG | 5.85 x10^5^ - 4560 | 0.990 | -3.25 | Putative Dam methylase |
| CM2 qPCR R | GTCGGCCTCAGTTAGC |
| CM5 qPCR F | TTATCACCGTCACAATTTGC | 7.12 x10^5^ - 1620 | 0.997 | -3.45 | Phage gene of unknown function |
| CM5 qPCR R | CTGCTTACACTGTAAGAACG |
| CM7 qPCR F | TATGGCCTATTCAGAGG | 3.55 x10^5^ - 1730 | 0.996 | -3.37 | Phage gene of unknown function |
| CM7 qPCR R | AACGACGTACTGTTATCC |
| CM18 qPCR F | TGAAAGCAACAGCACG | 3.55 x10^6^ - 1550 | 0.990 | -3.16 | Homologous to Lambda *lom* gene |
| CM18 qPCR R | CCAATGCCAGCAGTAACG |
