# Supplementary material for: Identification of genes expressed in cultures of E. coli lysogens carrying the Shiga toxin-encoding prophage Φ24B
Source: BMC Microbiol. 2012 Mar 22;12:42. doi: 10.1186/1471-2180-12-42 (PMC3342100; doi:10.1186/1471-2180-12-42)
Supplement: Additional file 2 — Table S2. Significance of Dunnett's test results for gene expression data in Figure 3: Results of the Dunnett's test to determine significance of gene expression profile differences before and after prophage induction. [file 1471-2180-12-42-S2.DOC]

**Supplementary Table 2. Significance of Dunnett’s test results for gene expression data in Fig. 3.**

| **Time point** | ***p*-value** | | | | | | | | | | |  |  |
| --- | --- | --- | --- | --- | --- | --- | --- | --- | --- | --- | --- | --- | --- |
|  | cI | Q | Cro | Ter | Capsid | GyrB | 16s | P1 | P2 | P3 | P4 | P5 | P6 |
| **0** | 0.013 | 1.000 | 0.997 | 1.000 | 1.000 | 0.717 | 0.084 | 1.000 | 0.183 | 0.341 | 1.000 | 1.000 | 1.000 |
| **10** | 0.999 | 1.000 | 0.996 | 1.000 | 1.000 | 0.753 | 1.000 | 0.960 | 0.879 | 0.272 | 0.645 | 1.000 | 1.000 |
| **20** | **0.002** | 0.999 | 0.994 | 1.000 | 1.000 | 1.000 | 0.772 | 0.999 | 0.171 | 0.185 | 0.485 | 1.000 | 1.000 |
| **30** | 0.097 | 0.085 | 0.076 | 0.999 | 0.999 | 0.230 | 0.285 | 0.241 | 0.163 | 0.193 | 0.065 | 0.904 | 0.972 |
| **40** | **0.007** | 0.097 | **0.003** | 0.805 | 0.995 | 1.000 | 0.138 | 0.475 | 0.163 | 0.204 | 0.437 | 0.125 | 1.000 |
| **50** | **0.012** | **0.006** | **0.007** | **0.002** | **0.007** | 0.998 | 0.121 | **0.035** | 0.170 | 0.201 | 0.945 | **0.001** | 0.270 |
| **60** | **0.003** | 0.789 | 0.796 | 0.293 | 0.890 | 0.731 | 0.080 | 0.998 | 0.179 | 0.184 | 0.922 | 1.000 | 1.000 |

| **Time**  **point** | ***p*-value** | | | |  |
| --- | --- | --- | --- | --- | --- |
|  | CM1 | CM2 | CM 5 | CM7 | CM18 |
| **0** | 1.000 | 0.912 | 1.000 | 0.999 | 0.225 |
| **15** | 1.000 | 1.000 | 1.000 | 0.999 | 0.727 |
| **30** | 1.000 | 1.000 | 1.000 | 0.999 | 0.603 |
| **45** | 0.114 | **0.002** | 0.594 | **0.000** | 0.894 |
| **60** | **0.000** | **0.001** | **0.001** | **0.000** | 0.324 |
